# Toxicological Profile of the Aqueous Extract of *Tectona grandis* L.F. (Verbenaceae) Leaves: A Medicinal Plant Used in the Treatment of Typhoid Fever in Traditional Cameroonian Medicine

**DOI:** 10.1155/2021/6646771

**Published:** 2021-04-05

**Authors:** Gabriel Tchuente Kamsu, Dieudonné Pascal Djamen Chuisseu, Siméon Pierre Fodouop Chegaing, Huguette Bocanestine Laure Feudjio, Louis-Claire Ndel Famen, Norbert Kodjio, Jean Baptiste Sokoudjou, Donatien Gatsing

**Affiliations:** ^1^Department of Biochemistry, Faculty of Science, University of Dschang, P.O. Box 67, Dschang, Cameroon; ^2^Department of Basic Sciences, Higher Institute of Health Sciences, Université des Montagnes, P.O. Box 208, Bangangté, Cameroon; ^3^Department of Biomedical Sciences, Faculty of Medicine, University of Ngaoundéré, P.O. Box 454, Ngaoundéré, Cameroon

## Abstract

*Tectona grandis* (*T. grandis*) is a medicinal plant widely used in Cameroon to treat typhoid fever and several other diseases. Despite its heavy use for medical purposes, no study has yet been conducted to assess its potentially toxic effects. This study aimed at evaluating the acute and subchronic toxicological profile of *Tectona grandis* leaf extract in rats. The acute toxicity study revealed neither behavioral disturbances nor death in rats. The lethal dose (DL_50_) of this extract is greater than 5000 mg/kg body weight. The subchronic toxicity study showed no significant change in weight gain in rats at test doses throughout the treatment period. However, there was a significant decrease in alanine transaminase activity and serum protein levels at all doses. Alkaline phosphatase activity decreased at doses of 30, 90, and 270 mg/kg and increased at the dose of 810 mg/kg body weight. Serum and urinary urea levels increased simultaneously at doses of 270 and 810 mg/kg body weight. Repeated administration of the extract also increased total cholesterol, high-density lipoprotein levels in both sexes were compared to respective controls, and the ratio of high- to low-density lipoprotein was found to be greater than 1 in all animals. However, at the dose of 810 mg/kg, necrosis was observed on the kidney sections and vascular congestion on the liver sections of animals. Aqueous extract of *T. grandis* did not lead to any adverse effects in rats after acute and subchronic treatment at 30 and 90 mg/kg doses. This extract can, therefore, be used for the formulation of typhoid fever phytomedicine at the therapeutic dose of 30 mg/kg, but before this, chronic and mutagenic toxicity evaluations must be carried out.

## 1. Background

Many research works have recognized the beneficial effects of medicinal plants on health as well as their capacity to prevent many diseases [1]. The use of medicinal plants which are readily available and efficacious would, therefore, be a better and affordable alternative for boosting and enhancing health [2]. However, the inadequate quality control, efficacy, and safety validation of medicinal plants has raised concerns over the last decades [3]. These intoxications are generally due to toxic compounds such as pyrrolizidine, vicine, andromedotoxin, and 5-hydroxylapachol [4], whose presence in these plants can irreversibly damage the liver, kidneys, and blood cells [5]. Several studies have shown the harmful effects of plant extracts, such as *Hibiscus rosa-sinensis*, *Bougainvillea spectabilis*, and *Cassia occidentalis* [6, 7]. Therefore, safety/toxicity evaluation of these plants such as *Tectona grandis* using animal models is widely encouraged, since the responses of these animals to chemical agents could be translated to humans.

*Tectona grandis* is a plant belonging to the Verbenaceae family, which is commonly found in India and tropical countries, and well known as “Teak.” The different parts (leaves, bark, and root [8, 9]; heartwood [10]; root heartwood [11]) of this plant contain many secondary metabolites which justify its traditional use as an anti-inflammatory, laxative, astringent, and analgesic plant [12]. In Cameroon, it is traditionally used in the Moungo Department (Loum) to treat diabetes and typhoid fever, and its *in vitro* and *in vivo* antityphoid activity has recently been proved [13]. Compounds isolated from *Tectona grandis* leaves have many biological activities: naphthotectone, anthratectone, and tectoionol A have allelopathic activity [14], while tectograndone, grandiquinone A, and hydroxysesamone have antibacterial and antimalarial activities [15]. Previous toxicological studies showed that the methanolic seed and leaf extract of *T. grandis* is nontoxic after single orally administration to albino mice [16, 17]. Knowing that the chemical composition and thus the activity/toxicity of a plant may vary depending on the harvested site and type of extracting solvent, it is important to study the potential side effects that the consumption of the “Cameroonian” species of *T. grandis* can cause to the consumer after single and repeated administration.

## 2. Materials and Methods

### 2.1. Plant Material

The leaves of *Tectona grandis* were harvested in Loum (Moungo Division, Littoral Region of Cameroon) in August 2018. The plant was identified at the National Herbarium of Cameroon in comparison with the reference sample kept under number 18580/SRF Cam (D. Dang Botanical Collection No. 160 Ref.).

### 2.2. Preparation of Extract

The leaves of *Tectona grandis* were harvested, dried out in the sun (about 25°C), and crushed. The obtained powder was used for the preparation of aqueous extract (Decoction) using the method described by Duke [18]. To do this, 500 g of powder was introduced into 5 L of distilled water and brought the mixture to boil for 15 minutes. After cooling, the mixture was filtered with Whatman N°1 paper, and the filtrates were dried in a ventilator oven (Memmert) at 40°C until the solvent had evaporated completely. The extracts obtained were kept away from light and stored at 4°C.

### 2.3. Experimental Animals

Wistar albino rats were used in this study. They were bred in the animal house of the Department of Biochemistry, University of Dschang, Cameroon. The rats were housed individually in polypropylene cages at 23 ± 1°C in a 12 h: 12 h dark: light cycle. The animals were provided with a standard diet and water *ad libitum,* and the food was withdrawn 12 h before the start of the experiment. This work was carried out with respect to the welfare of animals, as recommended by the Institutional Ethical Review Committee of the University of Dschang-Cameroon. Ethical guidelines and procedures for handling experimental animals were followed.

### 2.4. Acute Toxicity Study

Acute toxicity study was conducted under the OECD guideline with nine healthy females rats (age: 8 weeks; mass: 150–155 g), which were nulliparous and nonpregnant [19] and were randomly divided into three groups of three rats per group. Group I was the control group, and groups II and III received, respectively, a dose of 2000 and 5000 mg/kg of *T. Grandis* one after the other, and in a time interval of 48 hours. Each animal received the extract by gavage using an endogastric tube. After the survival of the three rats in series at the 2000 mg/kg dose, the 5000 mg/kg dose was administered to the rats. The trial ended when three serial rats survived to a limited dose of 5000 mg/kg.

After the administration of the test extract, mortality and clinical signs were noted for the first 3 hours and thereafter for 14 days of drug administration. During the 14 days, many physical symptoms of toxicity have been evaluated [20]. After 14 days of observation, each animal was autopsied, and eventual macroscopic changes were observed in organs such as the liver, kidneys, lungs, and heart.

### 2.5. Subchronic Toxicity

A 28-day subchronic toxicity study was conducted according to the standard methods [21, 22] with forty (40) rats (20 males and 20 females; age: 8-9 weeks; mass: 180–200 g) randomly assigned to 5 groups of 4 rats per group and sex. These rats received by endogastric gavage aqueous extract of *T. grandis* at 30 mg/kg, 90 mg/kg, 270 mg/kg, and 810 mg/kg doses of body weight for 28 days in batches 1, 2, 3, and 4, respectively. Batch 5 was the control group for each sex. Food consumption and body weight were determined daily for 28 days as described by Kamsu et al. [22]. On the 28^th^ day of the test, after gavages, the animals were subjected to a 12-hour food fast at the end of which urine was collected. Subsequently, the animals were anesthetized with diazepam/ketamine (0.2/0.1 ml per 100 grams of the animal) association, and the blood was collected by cardiac puncture.

### 2.6. Hematological Analysis

After the puncture, a small amount of blood was then introduced into a sterile tube containing an anticoagulant (EDTA) and was immediately used to perform a blood count using an impedance hematology automaton (QBC Autoread Plus, UK).

### 2.7. Biochemical Analysis

For biochemical analysis, blood samples were centrifuged at 3000 ×g for 10 min at 4°C. The serum was separated from the blood after centrifugation and stored at −20°C until analysis. Biochemical parameters like serum glutamic oxaloacetic transaminase (AST), glutamate-pyruvate transaminase (ALT), alkaline phosphatase (ALP), total protein, serum and urine creatinine, blood and urine urea, total cholesterol, triglyceride, and HDL cholesterol content were assayed using commercial kits (Spinreact, S.A.U Ctra., Santa Colona, Spain).

### 2.8. Histopathological Analysis

Histopathological *analysis* was performed on liver and kidney tissue sections. After euthanasia, all animals were autopsied and the major organs like the liver and kidney were surgically taken out and were fixed in 10% formalin in normal saline. Sections of 5 *μ*m were obtained on a rotary microtome, and then, the material was stained by hematoxylin-eosin (HE) [23]. The stained slides of the sections of the 40 tested animals were then analyzed using a microscope with an integrated digital photo camera (EVOS XL, USA) under an objective magnification of ×40 for possible anomalies. The histology of the treated groups was compared to the histology of the control group. After examination, photomicrographs of the liver and kidneys selected and presented in the document represent the general appearance observed in at least three of the four animals in the group.

### 2.9. Statistical Analysis

Statistical analysis was performed using SPSS version 23.0 for Windows. The results were expressed as mean value ± standard deviation (SD), and the comparisons were performed by the analysis of variance using a one-way ANOVA test. Differences between the averages of control and drug-treated groups where they existed were separated using the Waller–Duncan test. *p* value less than 0.05 was considered statistically significant.

## 3. Results

### 3.1. Acute Toxicity Study

**Figure 1:**
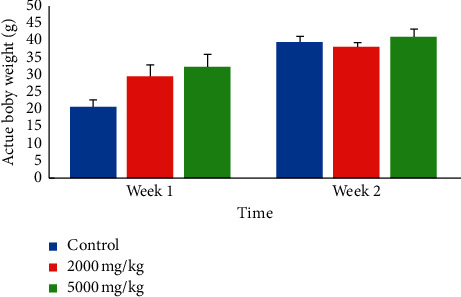
Evolution of body weight during acute administration of aqueous extract of *T. grandis* leaves. Data are expressed as mean ± SD, *n* = 4. Values for a given group in a column followed by a different letter as superscript are significantly different according to Waller–Duncan's multiple comparison test (*p* < 0.05).

Table 1 shows the behavior of the animals during the first three hours of observation following the administration of the aqueous extract of *T. grandis*. It can be seen that no death or signs of toxicity occurred in the animals in the groups when the extract was administered. The lethal dose (DL50) of this extract is greater than 5000 mg/kg body weight. Nevertheless, the animals given 5000 mg/kg of extract were calm for 2 hours after administration, although they were agitated afterward. No significant difference (*p* < 0.05) was observed in weight gain (Figure 1) and relative organ weights of the animals (Table 2). However, a dose-dependent increase in the weight of the animals was observed at week 1.

### 3.2. Subchronic Toxicity Study

#### 3.2.1. Evolution of Food Consumption

**Figure 2:**
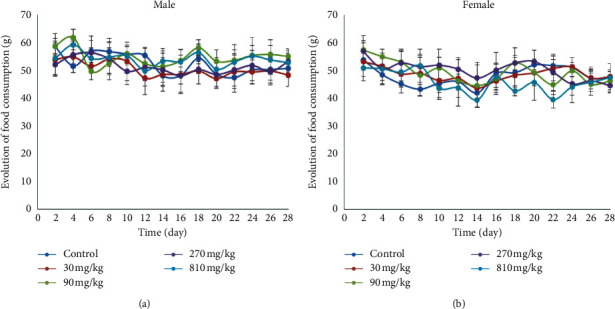
Effect of subchronic administration of aqueous extract of *T. grandis* on food consumption of rats. Data are expressed as mean ± SD, *n* = 4. Values for a given group in a column followed by a different letter as superscript are significantly different according to Waller–Duncan's multiple comparison test (*p* < 0.05). (a) Males. (b) Females.

Figure 2 shows the evolution of feed consumption of the animals during the 28 days of administration of the different doses of the extract. It can be seen that repeated administration of the aqueous extract of *T. grandis* did not produce any significant difference in the food consumption of male and female rats compared to the control animals.

#### 3.2.2. Evolution of Body Weight

**Figure 3:**
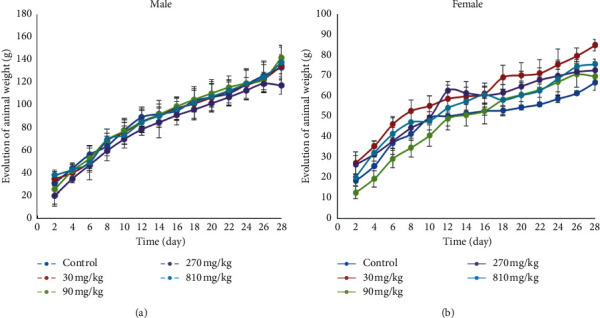
Effect of subchronic administration of aqueous extract of *T. grandis* on body weight of rats. Data are expressed as mean ± SD, *n* = 4. Values for a given group in a column followed by a different letter as superscript are significantly different according to Waller–Duncan's multiple comparison test (*p* < 0.05). (a) Males. (b) Females.

Figure 3 shows the evolution of feed consumption of the animals during the 28 days of administration of the different doses of the extract. This figure shows a nonsignificant increase in body weight of both sexes at all doses used during the 28-day test compared to the control.

#### 3.2.3. Changes in Relative Organ Weight

The effect of the aqueous extract from the leaves of *T. grandis* on the relative organ weights of the animals after 28 days of administration is shown in Table 3. It shows that no significant changes were observed in liver, kidney, spleen, lung, heart, and gonad weights at the end of 28 days of administration of the extract compared to the animals in the control group.

#### 3.2.4. Changes in Hematological Parameters

Table 4 shows the effects of the aqueous extract of *T. grandis* on hematological parameters after 28 days of repeated administration. It shows that except for a significant (*p* < 0.05) decrease in hemoglobin levels at 90 and 270 mg/kg and hematocrit levels at 270 mg/kg in males, no significant changes in other hematological parameters were observed in animals of both sexes at all doses of the extract when compared with animals in the control group.

#### 3.2.5. Changes in Biochemical Parameters

The effects of the extract on markers of liver function in animals after 28 days of repeated administration of different doses of *T. grandis* extract are presented in Table 5. The analysis of this table shows that repeated exposure of the animals to the extract resulted in a significant (*p* < 0.05) decrease in ALT activity and total serum protein levels in both sexes compared to control animals. Moreover, PAL activity decreased significantly (*p* < 0.05) at 30, 90, and 270 mg/kg in males and 30 and 90 mg/kg in females. However, ALP activity increased significantly (*p* < 0.05) at 810 mg/kg in both males and females compared to the control. The AST activity in animals of both sexes showed no significant difference.

The effect of different doses of *T. grandis* extract on markers of renal function in animals after 28 days of administration is presented in Table 6. These results show a significant (*p* < 0.05) increase in serum and urine urea levels at 270 and 810 mg/kg in animals of both sexes when compared to animals in the control group. However, serum creatinine levels decreased (*p* < 0.05) in animals of both sexes when compared to the control animals, while urinary creatinine and urinary protein levels remained statically unchanged.

The effect of the extract on the lipid profile of the animals is presented in Table 7. The analysis of this table shows that repeated exposure of the animals to different doses of the extract significantly (*p* < 0.05) increased the levels of total cholesterol, HDL cholesterol, and LDL cholesterol (except at the dose of 90 mg/kg in males) in animals of both sexes compared to animals in the control group. There was also a significant increase (*p* < 0.05) in triglyceride levels at 90 and 270 mg/kg in males and at test doses in females, except at 90 mg/kg, compared to animals in the control group. The HDL/LDL ratio was greater than 1 in all animals.

#### 3.2.6. Histological Sections of the Liver and Kidneys

**Figure 4:**
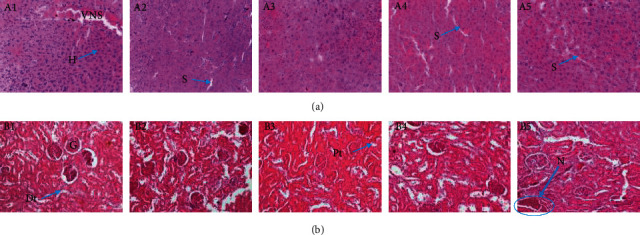
Effects of aqueous extract of *T. grandis* leaves on histological features of the liver (a) and kidney (b) in female rats (H&E × 400). A1, B1: control; A2, B2: 30 mg/kg; A3, B3: 90 mg/kg; A4, B4: 270 mg/kg; A5, B5: 810 mg/kg. G = glomerulus; N: necrosis; VNS: transected portal vein; S: sinusoids; H: hepatocytes; Pt: proximal tube; Dt: distal tube. The liver and kidney photomicrographs presented in the document represent the general appearance observed in at least three of the four animals in each group.

**Figure 5:**
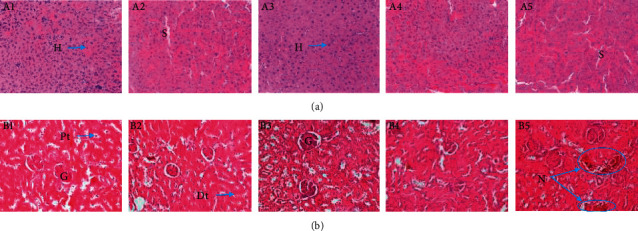
Effects of aqueous extract of *T. grandis* leaves on histological features of the liver (a) and kidney (b) in male rats (H&E × 400). A1, B1: control; A2, B2: 30 mg/kg; A3, B3: 90 mg/kg; A4, B4: 270 mg/kg; A5, B5: 810 mg/kg. G: glomerulus; N: necrosis; S: sinusoids; H: hepatocytes; Pt: proximal tube; Dt: distal tube. The liver and kidney photomicrographs presented in the document represent the general appearance observed in at least three of the four animals in each group.

Repeated administration of the aqueous extract of *T. grandis* for 28 days did not cause adverse effects on liver and kidney tissues of rats tested at doses of 270 mg/kg or less (Figures 4 and 5). However, at a dose of 810 mg/kg, necrosis was noted on the renal sections of both sexes.

## 4. Discussion

The toxicological profile of a substance is useful in the assessment of risks to humans. However, adverse changes in animals have a strong predictive value for human toxicity. When a substance is toxic, the main targets are the liver, kidneys, hematopoietic cells, and blood vessels [5]. For this reason, an assessment of the levels of markers of the correct functioning of these tissues can reflect the degree of toxicity of a substance. African and Asian traditional medicine has used the *T. grandis* plant for centuries for the treatment of various diseases, without scientific evidence of its safety. It is in this logic of having an idea about its harmlessness that this work was carried out. Thus, this work showed that the aqueous extract of the leaves of *T. grandis* showed no signs of toxicity, even at a dose of 5000 mg/kg in a single administration. The slight increase in weight gain observed in the first week is due to the increased feed intake of these animals to counter the action of the high doses of the extract. As no deaths were observed throughout the experiment, the LD_50_ of the aqueous extract of *T. grandis* is, therefore, higher than 5000 mg/kg. Thus, the leaves of *T. grandis* can be considered nontoxic for their traditional use, according to the Hodge and Steiner toxicity scale [24, 25]. This result corroborates the work of Dokuparthi et al. [26], who showed that the methanolic extract of *T. grandis* seeds is harmless at a dose of 5000 mg/kg in albino mice.

Repeated administration of the aqueous extract from the leaves of *T. grandis* at different doses for 28 days showed no variation (*p* < 0.05) in weight gain and food consumption. This result could reflect the fact that the feed consumption of treated animals and their weight growth evolved simultaneously, which would reflect a direct effect of feed consumption on the weight gain of animals in the test and control groups of both sexes. This extract does not cause anorexia in animals. Indeed, the absence of tannins in this extract [13], known for their anorexic and antinutritional properties, can confirm these results.

Hematopoiesis is a vital process for life and can be affected by both conventional and herbal medicines. Evaluation of hematological parameters in animal models provides information on the blood benefits of plant extracts and on their toxic manifestations [27]. The results of the hematological evaluation in this study did not show significant changes in hematological parameters. However, the significant decrease (*p* < 0.05) in hematocrit and hemoglobin levels observed in males at 90 and 270 mg/kg may be due to hyperhydration in the animals and not anemia, as no significant difference was observed in red blood cell levels. No significant changes were observed in total white blood cells and their differentials. As these cells are the main effectors of innate and adaptive immunity [28, 29], it can be deduced that the extract has no effect on immune responses. The number of platelets has also not been altered by the extract at all doses, implying that the leaf extract has no effect on hemostasis, which is controlled by platelets. All observations indicate the nonhematotoxic nature of the extract at the doses used in this study. Similar effects have been observed before with ethanol extracts of *Lychnophora pinaster* and *Anthonotha macrophylla* [30, 31].

The significant decrease (*p* < 0.05) in ALT activity at all doses and in ALP up to 270 mg/kg in animals of both sexes reflects the protective effect of our extracts at these doses on the liver. Indeed, the extract would act by reinforcing the selective permeability of the liver cell membranes, thus leading to a decrease in the level of serum enzymes and proteins. As ALT is an enzyme essentially limited to the liver and, therefore, considered a major indicator of hepatotoxicity [22, 32], the decrease in its serum concentration is an advantage for the liver. The absence of variation in the activity of AST shows, in addition to the harmlessness on the liver, the harmlessness of *T. grandis* extract on the muscles. ALP is present in the liver, bone, heart, skeletal muscle, kidneys, brain, pancreas, and blood cells [33]. The significant increase (*p* < 0.05) in ALP activity at 810 mg/kg in males and females may be due to altered hepatobiliary function or may be due to unknown tissue damage (cytolytic effects) at this dose caused by certain compounds present in the extract. Increased ALP activity is then a distinctive feature of cholestasis characterized by insufficient biliary excretion [34]. However, histology of the liver revealed no abnormalities.

The kidneys are the main route of excretion of drugs and their metabolites and are, therefore, exposed to high concentrations of potentially toxic substances often contained in drugs. Urinary protein levels and the concentration of urea and creatinine in serum and urine are used as a first-line measure of kidney function [35]. The simultaneous increase in serum urea levels from 90 mg/kg and urinary urea levels from 270 mg/kg will be either due to the high content of nitrogen compounds in the extract or due to the abundance of excreted nitrogen metabolites, as urea results from the metabolic processes of the ornithine cycle and is the main metabolic pathway for excretion of excess body nitrogen. During the same administration period, high concentrations (810 mg/kg) of this extract caused adverse effects (necrosis) on the kidneys. This could perhaps be due to the hyperactivity of toxic metabolites [11]. However, the variation in serum and urinary creatinine and urinary protein content is not very explicit here in the renal dysfunction observed in animals of both sexes.

Repeated administration of the aqueous extract from the leaves of *T. grandis* had no effect on the lipid profile of the animals. Indeed, according to Schaffer and Menche [36], excess “bad” cholesterol (LDL) and lack of “good” cholesterol (HDL) are risk factors for atherosclerosis. However, in this work, there is a simultaneous increase in both parameters, but the HDL/LDL cholesterol ratio being greater than 1 could exclude the risk of atherosclerosis and, therefore, toxicity. Thus, when the HDL cholesterol/LDL ratio is less than “1,” the risk of atherosclerosis is high and, therefore, indicates toxicity, when the HDL cholesterol/LDL ratio is between “1” and “2,” the extract has a nonsignificant beneficial effect on the lipid profile, and when the HDL cholesterol/LDL ratio is greater than “2,” the extract could have a significant beneficial effect. The aqueous extract from the leaves of *Tectona grandis* at a dose of 90 mg/kg could be described as significantly beneficial in males because the HDL/LDL cholesterol ratio is 8.34 ± 1.83.

## 5. Conclusion

The results suggest that acute administration of aqueous extract of *T. grandis* is associated with no signs of toxicity. During a long-duration administration, this extract does not present any risk of toxicity in rats at doses of 30 and 90 mg/kg but causes necrosis in kidneys at an 810 mg/kg dose. Further toxicological studies are required to evaluate the mutagenic, antimutagenic, and carcinogenic effects of this plant *in vitro* and *in vivo* as well as a chronic toxicity.

## Figures and Tables

**Table 1:** Effect of aqueous extract of *T. grandis* leaves on mortality and behavior of animals during acute administration.

Groups	Number deaths/total number	Sign of toxicity/behavioral changes
Control	0/3	No toxic effects
2000 mg/kg	0/3	No toxic effects
5000 mg/kg	0/3	Calms the first 2 hrs after administration, but agitated after

**Table 2:** Effect of aqueous extract of *T. grandis* leaves on relative organ weights of animals after 14 observation days.

Groups	Liver (g)	Kidneys (g)	Spleen (g)	Lungs (g)	Heart (g)	Gonads (g)
Control	4.09 ± 0.04^a^	0.72 ± 0.01^a^	0.31 ± 0.01^a^	0.58 ± 0.01^a^	0.30 ± 0.01^a^	0.03 ± 0.01^a^
2000 mg/kg	3.98 ± 0.14^a^	0.70 ± 0.07a^a^	0.30 ± 0.02^a^	0.59 ± 0.09^a^	0.29 ± 0.02^a^	0.03 ± 0.01^a^
5000 mg/kg	3.91 ± 0.49^a^	0.69 ± 0.08^a^	0.31 ± 0.02^a^	0.58 ± 0.08^a^	0.29 ± 0.02^a^	0.03 ± 0.01^a^

Data are expressed as mean ± SD, *n* = 3. Values for a given group in a column followed by a different letter as superscript are significantly different according to Waller–Duncan's multiple comparison test (*p* < 0.05).

**Table 3:** Effect of aqueous extract of *T. grandis* leaves on relative organ weights of animals.

Groups	Liver (g)	Kidneys (g)	Spleen (g)	Lungs (g)	Heart (g)	Gonads (g)
*Males*						
Control	3.20 ± 0.18^a^	0.64 ± 0.04^a^	0.27 ± 0.10^a^	0.51 ± 0.08^a^	0.27 ± 0.01^a^	0.92 ± 0.04^a^
30 mg/kg	3.10 ± 0.11^a^	0.63 ± 0.02^a^	0.30 ± 0.10^a^	0.50 ± 0.06^a^	0.29 ± 0.02^a^	0.97 ± 0.12^a^
90 mg/kg	3.09 ± 0.29^a^	0.62 ± 0.02^a^	0.29 ± 0.05^a^	0.51 ± 0.03^a^	0.28 ± 0.01^a^	0.96 ± 0.09^a^
270 mg/kg	3.45 ± 0.58^a^	0.68 ± 0.11^a^	0.37 ± 0.19^a^	0.57 ± 0.18^a^	0.31 ± 0.03^a^	0.96 ± 0.04^a^
810 mg/kg	3.08 ± 0.29^a^	0.65 ± 0.05^a^	0.20 ± 0.03^a^	0.43 ± 0.04^a^	0.28 ± 0.04^a^	0.92 ± 0.10^a^

*Females*						
Control	3.20 ± 0.41^a^	0.63 ± 0.07^a^	0.27 ± 0.09^a^	0.60 ± 0.09^a^	0.31 ± 0.03^a^	0.05 ± 0.01^a^
30 mg/kg	3.33 ± 0.25^a^	0.64 ± 0.05^a^	0.33 ± 0.07^a^	0.49 ± 0.04^a^	0.28 ± 0.03^a^	0.04 ± 0.01^a^
90 mg/kg	3.16 ± 0.24^a^	0.63 ± 0.04^a^	0.32 ± 0.08^a^	0.48 ± 0.09^a^	0.30 ± 0.02^a^	0.05 ± 0.01^a^
270 mg/kg	3.17 ± 0.10^a^	0.65 ± 0.04^a^	0.26 ± 0.06^a^	0.53 ± 0.05^a^	0.33 ± 0.05^a^	0.05 ± 0.01^a^
810 mg/kg	2.96 ± 0.10^a^	0.63 ± 0.04^a^	0.25 ± 0.10^a^	0.52 ± 0.08^a^	0.29 ± 0.02^a^	0.05 ± 0.01^a^

Data are expressed as mean ± SD, *n* = 4. Values for a given group in a column followed by a different letter as superscript are significantly different according to Waller–Duncan's multiple comparison test (*p* < 0.05).

**Table 4:** Effect of aqueous extract of *T. grandis* leaves on hematological parameters of animals.

Groups	RBCs (×10^6^/*μ*L)	HGB (g/dL)	HCT (%)	WBCs (×10^3^/*μ*L)	LYM (%)	MID (%)	GRAN (%)	PLT (10^3^/*μ*L)
*Males*								
Control	8.60 ± 0.41a	16.83 ± 0.55b	50.97 ± 1.69b	12.30 ± 0.96^a^	69.70 ± 9.19^a^	9.47 ± 3.74^a^	20.83 ± 5.46^a^	527.00 ± 101.95^a^
30 mg/kg	8.32 ± 0.53a	16.57 ± 0.95b	52.20 ± 3.92b	13.57 ± 1.42^a^	67.67 ± 0.74^a^	14.23 ± 0.50^a^	18.10 ± 1.21^a^	537.33 ± 133.72^a^
90 mg/kg	7.80 ± 0.19a	15.10 ± 0.36a	46.40 ± 2.76ab	13.07 ± 2.45^a^	65.37 ± 10.61^a^	10.53 ± 3.16^a^	24.10 ± 7.45^a^	438.33 ± 112.72^a^
270 mg/kg	7.98 ± 0.48a	15.00 ± 0.44a	43.83 ± 4.37a	13.10 ± 2.81^a^	61.17 ± 7.43^a^	13.57 ± 2.12^a^	25.27 ± 5.84^a^	486.67 ± 64.76^a^
810 mg/kg	8.23 ± 0.38a	16.00 ± 0.36ab	47.93 ± 2.41 ab	13.13 ± 0.75^a^	60.97 ± 4.00^a^	12.43 ± 0.50^a^	26.60 ± 3.50^a^	565.67 ± 47.61^a^

*Females*								
Control	7.67 ± 0.49^a^	15.57 ± 0.85^a^	47,40 ± 3,58^a^	9.43 ± 1.58^a^	66.77 ± 3.58^a^	12.37 ± 0.38^a^	20.87 ± 3.95^a^	551.00 ± 124.83^a^
30 mg/kg	7.83 ± 0.88^a^	15.60 ± 0.95^a^	48.37 ± 6.68^a^	12.20 ± 4.33^a^	75.87 ± 6.36^a^	10.70 ± 1.30^a^	16.43 ± 5.49^a^	467.67 ± 58.05^a^
90 mg/kg	7.88 ± 0.45^a^	15.60 ± 0.46^a^	46.90 ± 1.84^a^	11.03 ± 1.76^a^	69.40 ± 4.16^a^	10.40 ± 0.44^a^	20.20 ± 3.84^a^	549.00 ± 62.43^a^
270 mg/kg	8.08 ± 1.06^a^	15.53 ± 0.76^a^	47.77 ± 5.76^a^	11.33 ± 1.19^a^	72.60 ± 7.67^a^	10.57 ± 3.61^a^	16.83 ± 5.44^a^	653.00 ± 133.41^a^
810 mg/kg	7.84 ± 0.43^a^	15.43 ± 1.24^a^	47.70 ± 3.20^a^	11.80 ± 0.96^a^	69.80 ± 1.40^a^	11.70 ± 2.59^a^	18.50 ± 3.30^a^	740.67 ± 219.72^a^

Data are expressed as mean ± SD, *n* = 4. Values for a given group in a column followed by a different letter as superscript are significantly different according to Waller–Duncan's multiple comparison test (*p* < 0.05). RBCs: red blood cells; HGB: hemoglobin; LYM: lymphocytes; MID: mean corpuscular volume; GRAN: granulocytes; WBCs: white blood cells; PLT: platelets; HCT: hematocrit.

**Table 5:** Effect of aqueous extract of *T. grandis* leaves on liver function markers of animals.

Groups	ALT (U/L)	AST (U/L)	ALP (U/L)	Serum protein (mg/dL)
*Males*				
Control	354.15 ± 18.88^e^	30.41 ± 7.09^a^	130.35 ± 14.00^c^	6.73 ± 1.02^b^
30 mg/kg	227.93 ± 17.99^d^	30.63 ± 4.35^a^	122.51 ± 14.96^bc^	6.20 ± 1.54^b^
90 mg/kg	184.40 ± 16.77^c^	39.66 ± 3.66^a^	96.94 ± 9.17^a^	2.51 ± 0.91^a^
270 mg/kg	112.65 ± 6.90^b^	35.88 ± 8.60^a^	109.31 ± 7.90^ab^	2.71 ± 0.78^a^
810 mg/kg	83.34 ± 4.81^a^	36.09 ± 9.88^a^	166.66 ± 10.89^d^	2.08 ± 0.66^a^

*Females*				
Control	278.25 ± 15.27^c^	23.41 ± 3.94^a^	80.85 ± 6.17^ab^	5.74 ± 1.06^b^
30 mg/kg	107.84 ± 6.11^b^	24.06 ± 5.23^a^	63.94 ± 6.37^a^	3.13 ± 0.74^a^
90 mg/kg	70.87 ± 6.77^a^	27.41 ± 4.92^a^	68.89 ± 7.55^a^	3.08 ± 0.72^a^
270 mg/kg	99.09 ± 13.16^b^	26.25 ± 4.35^a^	98.59 ± 9.66^b^	4.32 ± 1.06^ab^
810 mg/kg	66.93 ± 13.02^a^	26.91 ± 3.31^a^	126.13 ± 16.42^c^	3.77 ± 0.85^a^

Data are expressed as mean ± SD, *n* = 4. Values for a given group in a column followed by a different letter as superscript are significantly different according to Waller–Duncan's multiple comparison test (*p* < 0.05). ALT: alanine transaminase; AST: aspartate transaminase; ALP: alkaline phosphatase.

**Table 6:** Effect of aqueous extract of *T. grandis* leaves on kidney function markers of animals.

Groups	S-Urea (mg/dL)	S-Crea (mg/dL)	U-Urea (mg/dL)	U-Crea (mg/dL)	Urine protein (mg/dL)
*Males*					
Control	73.50 ± 6.75^ab^	1.61 ± 0.51^b^	4145.83 ± 146.58^a^	7.73 ± 0.95^b^	0.36 ± 0.09^a^
30 mg/kg	62.83 ± 13.84^a^	1.44 ± 0.55^b^	3591.67 ± 46.39^a^	8.33 ± 1.68^b^	0.60 ± 0.52^a^
90 mg/kg	72.50 ± 8.24^ab^	1.11 ± 0.22^ab^	2612.50 ± 61.42^a^	7.14 ± 0.75^ab^	0.43 ± 0.23^a^
270 mg/kg	108.92 ± 10.12^c^	1.34 ± 0.16^ab^	8100.00 ± 115.31^b^	5.42 ± 0.88^a^	0.71 ± 0.40^a^
810 mg/kg	134.58 ± 18.55^d^	0.75 ± 0.19^a^	12283.33 ± 155.20^c^	9.39 ± 1.86^b^	0.52 ± 0.37^a^

*Females*					
Control	49.42 ± 11.85^a^	1.26 ± 0.37^b^	4333.33 ± 99.50^b^	7.14 ± 1.77^ab^	0.37 ± 0.10^a^
30 mg/kg	42.33 ± 3.19^a^	1.20 ± 0.25^b^	3341.67 ± 121.84^ab^	6.35 ± 1.99^ab^	0.58 ± 0.33^a^
90 mg/kg	50.50 ± 5.95^a^	0.86 ± 0.20^ab^	2812.50 ± 34.35^a^	5.75 ± 3.15^ab^	0.73 ± 0.44^a^
270 mg/kg	81.92 ± 15.04^b^	0.99 ± 0.35^ab^	4983.33 ± 62.31^b^	3.57 ± 0.82^a^	0.85 ± 0.11^a^
810 mg/kg	95.50 ± 10.99^b^	0.63 ± 0.04^a^	10158.33 ± 74.65^c^	7.67 ± 2.51^b^	0.47 ± 0.19^a^

Data are expressed as mean ± SD, *n* = 4. Values for a given group in a column followed by a different letter as superscript are significantly different according to Waller–Duncan's multiple comparison test (*p* < 0.05). S-Crea: serum creatinine, U-Crea: urine creatinine, S-Urea: serum urea, U-Urea: urine urea.

**Table 7:** Effect of aqueous extract of *T. grandis* leaves on lipid profile of animals.

Groups	Triglycerides (mg/dL)	T-cholesterol (mg/dL)	HDL (mg/dL)	LDL (mg/dL)	HDL/LDL ratio	Atherogenic index
*Males*						
Control	87.96 ± 3.05^a^	79.70 ± 6.57^a^	39.94 ± 7.10^a^	22.17 ± 7.29^ab^	2.09 ± 1.26^a^	1.03 ± 0.31^b^
30 mg/kg	87.04 ± 5.72^a^	116.34 ± 11.18^cd^	57.79 ± 14.58^ab^	41.14 ± 6.94^c^	1.46 ± 0.52^a^	1.08 ± 0.36^b^
90 mg/kg	104.63 ± 14.02^b^	89.72 ± 5.79^ab^	59.09 ± 14.01^ab^	9.70 ± 1.27^a^	8.34 ± 1.83^b^	0.54 ± 0.17^a^
270 mg/kg	117.59 ± 3.16^b^	123.05 ± 10.84^d^	67.42 ± 10.14^b^	32.11 ± 10.83^bc^	2.29 ± 0.78^a^	0.85 ± 0.29^ab^
810 mg/kg	84.48 ± 6.95^a^	100.00 ± 6.82^bc^	53.57 ± 6.55^ab^	29.53 ± 4.83^bc^	1.86 ± 0.42^a^	0.88 ± 0.17^ab^

*Females*						
Control	74.81 ± 6.23^a^	61.15 ± 7.20^a^	32.14 ± 2.63^a^	14.04 ± 7.75^a^	2.91 ± 1.52^b^	0.91 ± 0.24^a^
30 mg/kg	100.37 ± 7.37^b^	102.71 ± 14.19^b^	59.63 ± 8.17^b^	23.00 ± 2.99^ab^	2.71 ± 1.18^ab^	0.79 ± 0.31^a^
90 mg/kg	64.44 ± 5.41^a^	97.94 ± 10.96^b^	44.74 ± 1.87^ab^	40.31 ± 12.50^c^	1.20 ± 0.43^ab^	1.19 ± 0.28^ab^
270 mg/kg	91.30 ± 11.40^b^	86.36 ± 9.83^b^	36.26 ± 5.14^a^	31.85 ± 6.45^bc^	1.17 ± 0.27^a^	1.40 ± 0.33^b^
810 mg/kg	92.04 ± 4.93^b^	87.88 ± 4.08^b^	46.00 ± 6.49^ab^	23.48 ± 4.82^ab^	2.09 ± 0.84^ab^	0.93 ± 0.20^ab^

Data are expressed as mean ± SD, *n* = 4. Values for a given group in a column followed by a different letter as superscript are significantly different according to Waller–Duncan's multiple comparison test (*p* < 0.05). HDL: high-density lipoprotein; LDL: low-density lipoprotein; AI: Atherogenic index; T-Chol: total cholesterol.

## Data Availability

All data used and analyzed during the present study will be available from the corresponding author if deemed necessary.

## Abbreviations

*T. grandis*: *Tectona grandis*
ANOVA: One-way analysis of variance
SEM: Mean standard err.
H&E: Hematoxylin and eosin
OECD: Organization for Economic Co-operation and Development
RBCs: Red blood cells
HGB: Hemoglobin
LYM: Lymphocytes
MID: Mean corpuscular volume
GRAN: Granulocytes
WBCs: White blood cells
PLT: Platelets
HCT: Hematocrit
ALT: Alanine Transaminase
AST: Aspartate transaminase
ALP: Alkaline phosphatase
S-Crea: Serum creatinine
U-Crea: Urine creatinine
S-Urea: Serum urea
U-Urea: Urine urea
HDL: High-density lipoprotein
LDL: Low-density lipoprotein
AI: Atherogenic index
T-Chol: Total cholesterol.
